# Effects of Nutrient Heterogeneity and Competition on Root Architecture of Spruce Seedlings: Implications for an Essential Feature of Root Foraging

**DOI:** 10.1371/journal.pone.0065650

**Published:** 2013-06-06

**Authors:** Hongwei Nan, Qing Liu, Jinsong Chen, Xinying Cheng, Huajun Yin, Chunying Yin, Chunzhang Zhao

**Affiliations:** Key Laboratory of Mountain Ecological Restoration and Bioresource Utilization & Ecological Restoration Biodiversity Conservation Key Laboratory of Sichuan Province, Institute of Biology, Chinese Academy of Sciences, Chengdu, China; University of Michigan, United States of America

## Abstract

**Background:**

We have limited understanding of root foraging responses when plants were simultaneously exposed to nutrient heterogeneity and competition, and our goal was to determine whether and how plants integrate information about nutrients and neighbors in root foraging processes.

**Methodology/Principal Findings:**

The experiment was conducted in split-containers, wherein half of the roots of spruce (*Picea asperata*) seedlings were subjected to intraspecific root competition (the vegetated half), while the other half experienced no competition (the non-vegetated half). Experimental treatments included fertilization in the vegetated half (FV), the non-vegetated half (FNV), and both compartments (F), as well as no fertilization (NF). The root architecture indicators consisted of the number of root tips over the root surface (RTRS), the length percentage of diameter-based fine root subclasses to total fine root (SRLP), and the length percentage of each root order to total fine root (ROLP). The target plants used novel root foraging behaviors under different combinations of neighboring plant and localized fertilization. In addition, the significant increase in the RTRS of 0–0.2 mm fine roots after fertilization of the vegetated half alone and its significant decrease in fertilizer was applied throughout the plant clearly showed that plant root foraging behavior was regulated by local responses coupled with systemic control mechanisms.

**Conclusions/Significance:**

We measured the root foraging ability for woody plants by means of root architecture indicators constructed by the roots possessing essential nutrient uptake ability (i.e., the first three root orders), and provided new evidence that plants integrate multiple forms of environmental information, such as nutrient status and neighboring competitors, in a non-additive manner during the root foraging process. The interplay between the responses of individual root modules (repetitive root units) to localized environmental signals and the systemic control of these responses may well account for the non-additive features of the root foraging process.

## Introduction

Root foraging is one of the most important aspects of plant behavior because it can affect individual plant growth as well as plant fitness and community structure [1], [2]. The said process can respond to the presence of neighboring competitor roots and the heterogeneous distribution of nutrients in the soil [3], [4], particularly when the general levels of nutrient availability are low [5]–[7]. In nature, plants are simultaneously exposed to nutrient heterogeneity and the roots of neighbors. Recent studies reported that plant root growth could be an additive or a non-additive response to multiple forms of environmental information, which partially depends on the neighboring species or their competitive attributes [8]–[10]. Therefore, the incorporation of multiple simultaneous environmental conditions in root foraging studies may help to advance our understanding of the relationships between plant root systems and the environment.

In forest ecosystems, tree seedlings allow their roots to proliferate to acquire nutrients and water; seedlings typically contend with heterogeneous resources and competing neighbors, which both exert important effects on root foraging behavior [10], [11]. Previous forestry studies on root competition have invested much effort toward investigating the effects of interspecific competition [3], [12]–[14]; the importance of intraspecies interaction has received much less attention. Due to similarity in ecological characters, plants in intraspecies competition cannot avoid or alleviate adverse competition effect via niche complementarity. Accordingly, the thing missing from many studies of root competition is a detailed understanding of intraspecies interactions.

Root architecture is defined as the spatial configuration of the root system, which has a key role in belowground resource acquisition [15], [16]. Fitter et al. [17], [18], as well as Farley and Fitter [19], demonstrated that a herringbone topology may be best for locating nutrient-rich patches in the soil, but a less herringbone topology is more suitable for exploiting these resources. Grime and Mackey reported that phenotypic plasticity for specific root architectural traits was significant in resource capture, as a result of nutrient heterogeneity in space and time [20]. In addition, root architecture was shown to be a primary factor affecting the degree of competition among roots of the same plant and/or neighboring plants [21]–[23]. More recently, Nord et al. found that the presence of a neighbor could lead to alterations in the root architecture, thereby keeping the root biomass stable [24]. To date, accumulating evidence indicated that root architecture was more sensitive to environmental stimuli than root biomass [24], [25]. However, most studies addressing plant foraging ability have focused on root biomass but overlooked root architecture, which can contribute to a better understanding of the interactions between plant root systems and their environment.

Plant root foraging ability is closely related to root architecture, but none of the previous studies thus far have linked these aforementioned aspects of plant root systems. This oversight was probably because root functions, such as resource uptake and transport, were difficult to directly measure [26]. Previous studies mainly utilized lateral root attributes to assess the response of the root architecture to environmental stimuli; these attributes included descriptions of the morphological characteristics [27], [28], spatial deployment pattern [29], [30], and root-growth patterns [31]–[33]. However, all these measurements are unsuitable for precisely measuring the root foraging ability. In addition, the entire root system was traditionally divided into different parts based on size classes (e.g., 0–1 mm roots vs. 0–2 mm roots, based on their diameter), which did not provide information on the root system structure, function, and response to altered environmental conditions. This limitation is particularly true in woody plants because fine roots are complex branching structures composed of numerous individual root segments, which differ in their morphology and function. The position and form of individual roots on the branching fine root system are typically disregarded by the said classification modes [34]–[37]. Guo et al. examined the anatomy and mycorrhizal colonization of branch order in 23 Chinese temperate tree species, and demonstrated that active nutrient absorption was mainly achieved by the first three orders of the root system, particularly the first-order roots (tiny lateral branches at the very distal end of the root system) [37]. To effectively measure the root foraging ability, the first three root orders should collectively be taken into account, rather than the entire fine root system, when determining the root architecture indicators for woody plants. To the best of our knowledge, none of the previous studies have employed such novel indirect assessment methods of root foraging.

Plants producing preferentially roots in nutrient-rich substrate patches were proposed to function as the primary root foraging mechanism by which plants cope with the naturally occurring heterogeneous nutrient supply in soil [5], [38]. Several studies indicated that a plant in the presence of neighboring roots preferentially grows new roots in unoccupied soil before it does the same in a space already occupied by other species or conspecifics [21], [39]. However, little information is available on how the foraging behavior of plant root systems responds to the simultaneous presence of nutrient heterogeneity and neighboring roots [8], [10]. To obtain a more mechanistic understanding of plant root foraging response to neighbors and nutrients, we simultaneously manipulated nutrient heterogeneity and intraspecies competition conditions, investigated root foraging responses based on the root architecture, and assessed their influence on nutrient uptake in spruce (*Picea asperata*), the dominant tree species in the subalpine coniferous forests of western Sichuan, China.

## Materials and Methods

### Ethics Statement

The experiment was set up at an open field (31°25′N, 103°12′E, 2309 m, a.s.l.) in the Miyaluo natural reserve of Lixian County, Eastern Tibetan Plateau, in Sichuan, China. We obtained appropriate permissions from the Forestry Bureau of Lixian County, and from the forestry workers for field study. In present study, spruce (*P. asperata*) seedlings, the dominant tree species in natural reserve, were used as investigated subject, and we confirmed that our studies did not involve endangered or protected species. In addition, no specific permission was required for these locations because our study was the general pot experiment.

### Experimental Design and Treatments

The experimental site had a montane monsoon climate, which was humid and rainy in summer but cold and dry in winter, with mean January and July temperatures of −8°C and 12.6°C, respectively. The mean annual precipitation ranged from 600 mm to 1100 mm, and the mean annual evaporation was from 1000 mm to 1900 mm. The soil was classified as mountain brown earth [40].

**Figure 1:**
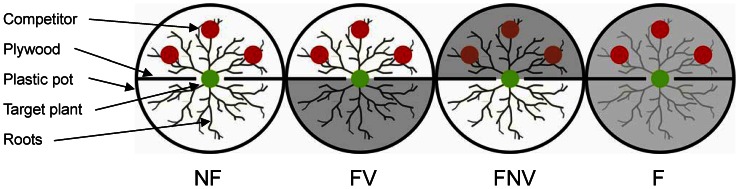
Schematic of the experimental treatments. The four treatments consisted of fertilization in the vegetated half (FV), the non-vegetated half (FNV), and both compartments (F), as well as no fertilization (NF).

On April 2011, 32 large circular plastic pots (38 cm in diameter, 30 cm deep) were divided into two parts of equal volume using solid plywood planks (see Fig. 1). The pots were filled with sieved, root free soil (4.5 mm mesh) from the neighboring forest. The basic soil properties were as follows: pH, 5.85; soil organic C, 62.70 mg·g^−1^; total N, 3.66 mg·g^−1^; total P, 0.43 mg·g^−1^; and total K, 7.92 mg·g^−1^.

At the beginning of May, three-year-old spruce (*P. asperata*) saplings of similar sizes were randomly established in the pots; the root systems of these saplings had nearly homogeneous and symmetrical distribution around the stem axis. One sapling to be used as the target plant was carefully placed in the middle of each pot. The main root of this sapling was then inserted into a narrow (3 cm) gap carved into the plywood plank, whereas the lateral roots were equally arrayed into separate compartments. Three spruce saplings were planted in half of each pot (the “vegetated half”) to function as competitors, whereas the other half (the “non-vegetated half”) had no saplings (Fig. 1).

In this study, all the four treatments were established by applying fertilizer in different compartments or otherwise. These treatments included fertilization in the vegetated half (FV), non-vegetated half (FNV), and both compartments (F), as well as no fertilization (NF); each treatment had eight pots. The fertilizer contained NPK in a 15∶1:1 ratio, based on Hoagland’s hydroponic solution [41]. The fertilizer was applied from June to mid-September at 1.0 g N·m^−2^ every 10 days (a total of ten times throughout the growing season).

### Root Measurements

In mid-September, all the target plant seedlings were carefully harvested by hand with the help of a watering hose, taking care to maintain the integrity of the root systems. Roots were then separated from each seedling and divided into two groups (without including the main root) based on the compartment where they were grown. All the root systems in each group were carefully washed free of soil. Their length, surface area, volume, and number of tips were measured using the WinRHIZO image analysis software (Régent instruments, Quebec, QC, Canada). In order to obtain more accurate morphological results, we scanned all the root systems, which were time- and energy-consuming, unlike previous studies that merely selected a few root samples per plant. Subsequently, three root samples per plant and compartment were chosen from the scanned roots. Each of the said root samples contained at least eight intact distal root segments, including more than three root orders. The samples were dissected to obtain the first three root orders using scalpel blades in large petri dish. The most distal root tips were classified as the first-order roots, whereas the second- and third-order roots were dissected according to the order of streams in geography [34]. The root morphologies of the first three root orders, such as the length and surface area, were assessed using the same image analysis software as mentioned above to determine the length and surface area ratio among the first three orders. Finally, all the root systems per plant and compartment were divided into two groups according to their diameter (fine roots, ≤2 mm, other roots, >2 mm). Their biomass was measured using a digital balance after drying in an oven at 70°C for 48 h. When the total biomass of fine roots per plant and compartment was calculated, the root biomass of the first three orders was then added to obtain the final value. Furthermore, the main roots and shoots of each seedling were washed carefully, and their biomass was measured using a similar method to determine the whole plant biomass.

A root architecture indicator defined in our study is the number of root tips over root surface (RTRS). More than 96% of the root tips were located in 0–0.5 mm fine roots, as demonstrated in our previous work. Thus, this region of the root surface alone was used for calculating the RTRS values to avoid errors. To further investigate the root architecture, we divided 0–0.5 mm fine roots into two subclasses based on their diameter, namely, the 0–0.2 mm and the 0.2–0.5 mm root systems. The RTRS of these subclasses could be calculated using of the above mentioned root morphology measurements.

Another root architecture indicator was the length percentage of diameter-based fine root subclasses to the total fine root length (subclass root length percentage, SRLP). Given that the first three orders in the root systems were the primary parts involved in nutrient absorption [37] and constituted the main body of 0–0.5 mm fine roots (average root diameter of the third-root order was approximately 0.46 mm in our study), we divided the whole fine root (≤2 mm) into three subclasses based on their diameter: the 0–0.5 mm, 0.5–1.0 mm, and 1.0–2.0 mm subclasses [42]. A high SRLP value of the 0–0.5 mm root system indicated more efficient root foraging ability, which could be calculated from root morphology measurements, as mentioned above.

To improve our understanding of the mechanisms involved, we determined the length percentage of each root order to the total fine root length as another indicator of the root architecture (root order length percentage, ROLP). The surface area and length of the first-order root were analyzed using the link analysis tool provided by WinRhizo™ 2009. According to Pregitzer’s definition [34], the first-order roots consisted of the external-external and the external-internal links; the morphological parameters of the first-order roots were equal to the sum of both links [43]. The root morphology of the second- and third-order was calculated using the morphology ratio among the first three orders, as described above. Based on these results, the ROLP could be calculated.

The biomass of the first three root orders, as described above, was a suitable indicator to assess the root foraging ability, except for the root architecture. We had acquired the respective length and surface area of the first three orders in the preceding methods, but their volumes remained unknown. Based on the morphological measurements of the abovementioned root samples, the respective regression of the volume on surface area for the first three orders was established. The respective volume of the first three orders per plant and compartment were calculated. Given the strongly linear relationship between the fine root volume and its biomass [44], the respective biomass of the first three orders were calculated using Cheng’s formula based on their volumetric percentage to that of the fine roots (≤2 mm) [42].

### Growth Measurement

Each target plant seedling was tagged when they were planted in early May. The basal diameter of each seedling was measured and recorded. Another fifteen additional spruce seedlings with similar sizes to the planted target seedlings were selected. Their basal diameters and whole plant biomass weights were simultaneously measured to establish the regression model of the whole plant biomass according to the basal diameter. Based on the regression equation, the initial biomass for each of the target plant seedlings was calculated. At the end of the growing season, the final plant biomass harvested was measured, as described above.

The relative growth rate (RGR) for each plant was calculated using the formula RGR = [ln *w*_2_– ln *w*_1_]/*T* [45]; where *w*_2_ and *w*_1_ are the final and initial plant biomass, respectively, whereas *T* is the number of months between the initial and final measurements (i.e., 3.5 months).

### Statistical Analyses

The root response was evaluated for each pot using the ratio between the root variable values in the vegetated and non-vegetated halves (e.g., RTRS _ratio_ = RTRS_ vegetated half_/RTRS _non-vegetated half_). The values of the root variables were considered to be higher in the non-vegetated half when the ratio was significantly lower than 1, and lower when the ratio is higher than 1 (i.e., the ratio is equal to 1 for symmetrical root growth). This difference was analyzed using a paired-sample *t*-test. Furthermore, the effects of different treatments on the root architecture and biomass in the vegetated and non-vegetated regions as well as the relative growth rate (RGR) were examined using factorial ANOVA for a randomized block design, with treatments as the fixed factors. The root biomass, architecture, and relative growth rate were recorded as dependent variables. The data were transformed when necessary using the natural logarithmic transformation to satisfy the normality and homogeneity of the variances. The overall data was statistically analyzed using the SPSS program (SPSS 13.0, Chicago).

## Results

### The First Three Order Root Biomass

**Figure 2:**
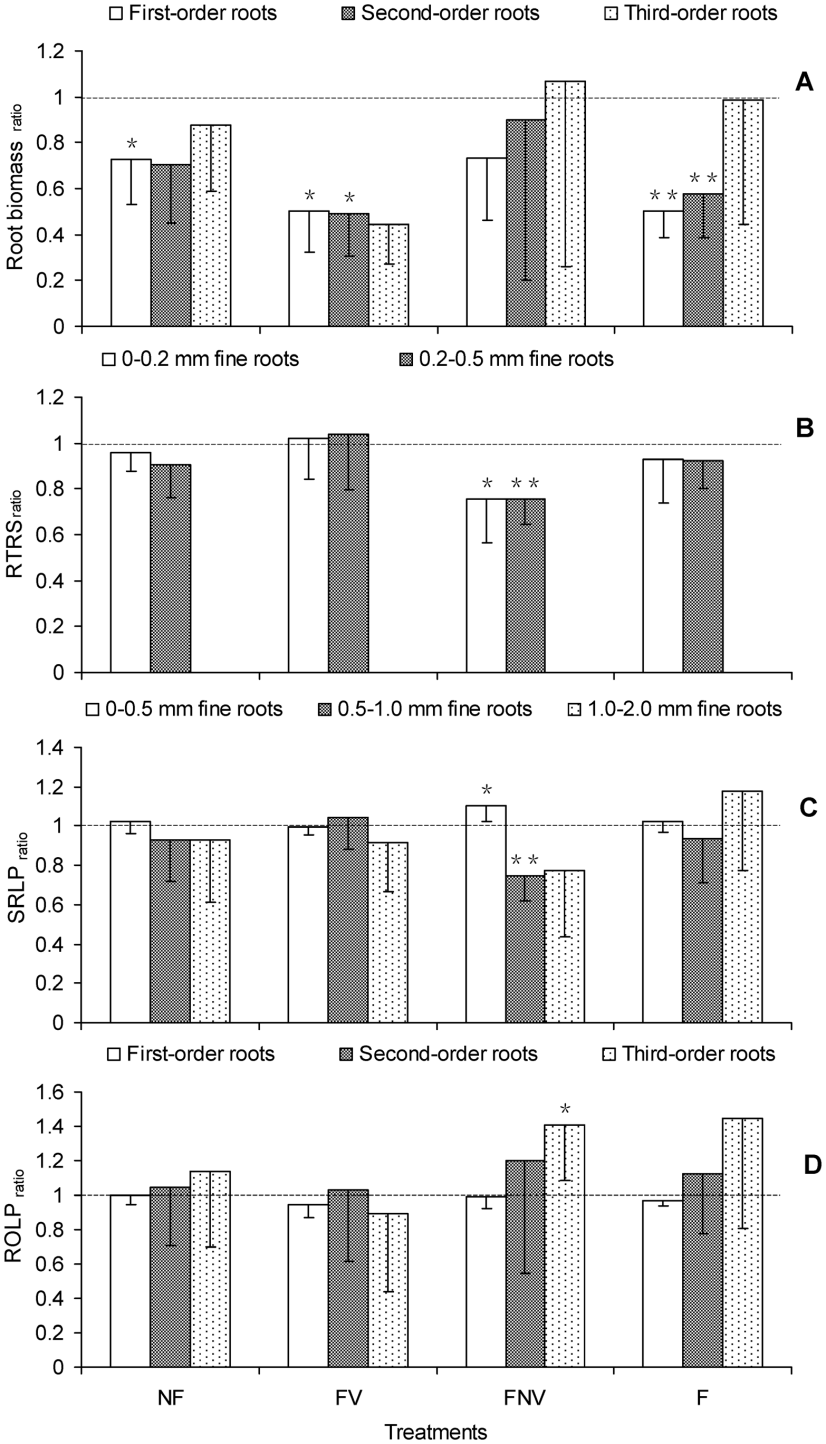
The ratio “vegetated half: non-vegetated half” in root system biomass and architecture. (A) root biomass ratio; (B) the number of root tips over the root surface ratio (RTRS _ratio_); (C) the length percentage ratio of diameter-based fine root subclasses to the total fine root length (SRLP _ratio_); (D) the length percentage ratio of each root order to the total fine root length (ROLP _ratio_). Asymmetrical root biomass and architecture (i.e. ratios significantly different from 1.0) are indicated above the columns (**P<0.01, *P<0.05). Error bars represent one SE of the mean.

**Figure 3:**
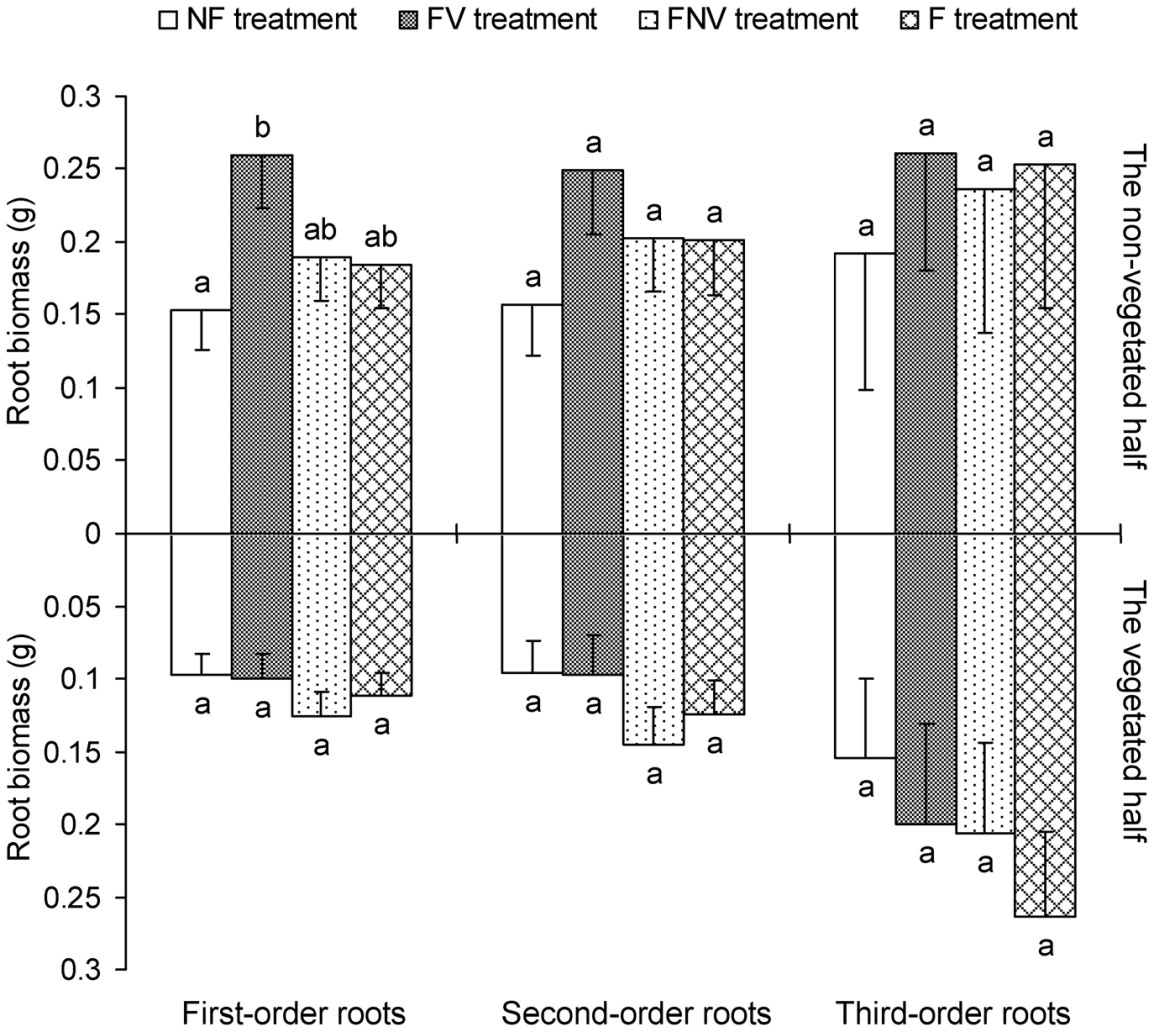
Root system biomass in the vegetated half and in the non-vegetated half. Letters indicate the same root order difference between treatments (LSD tests, following ANOVA). Error bars represent 1 SE of the mean.

The first three root orders were the most important sections of fine-root systems for nutrient and water acquisition. For woody plants with complicated branching order root systems, the fine root (≤2 mm) biomass was not suitable for measuring the root foraging ability. The first-order roots in the NF treatment, as well as the first- and second-order roots in both FV and F treatments, showed significantly lower root biomass ratios (i.e. ratios were significantly less than 1), whereas no significant differences were found for the third-order roots in all the four treatments, as well as in the first three root orders of the FNV plants (Fig. 2). These results indicated that except for the FNV treatment, root competition reduced the absorbing root biomass in the vegetated half of the target plant, which were mainly concentrated on the first two root orders. Furthermore, different root order responses were observed for various forms of root competition. The root biomass ratio in FNV treatment may have not been significantly different from 1 because the absorbing root biomass decreased as the soil resources were increased by the increased use of fertilizers in the non-vegetated half. In addition, the first-order root biomass significantly varied among the non-vegetated halves of the FV and NF treatments. By contrast, the biomass was not significantly different between the second- and third-order roots from the non-vegetated halves of all four treatments. No significant differences were observed among treatments in the vegetated half (Fig. 3).

### Root Architecture Indicator: RTRS

**Figure 4:**
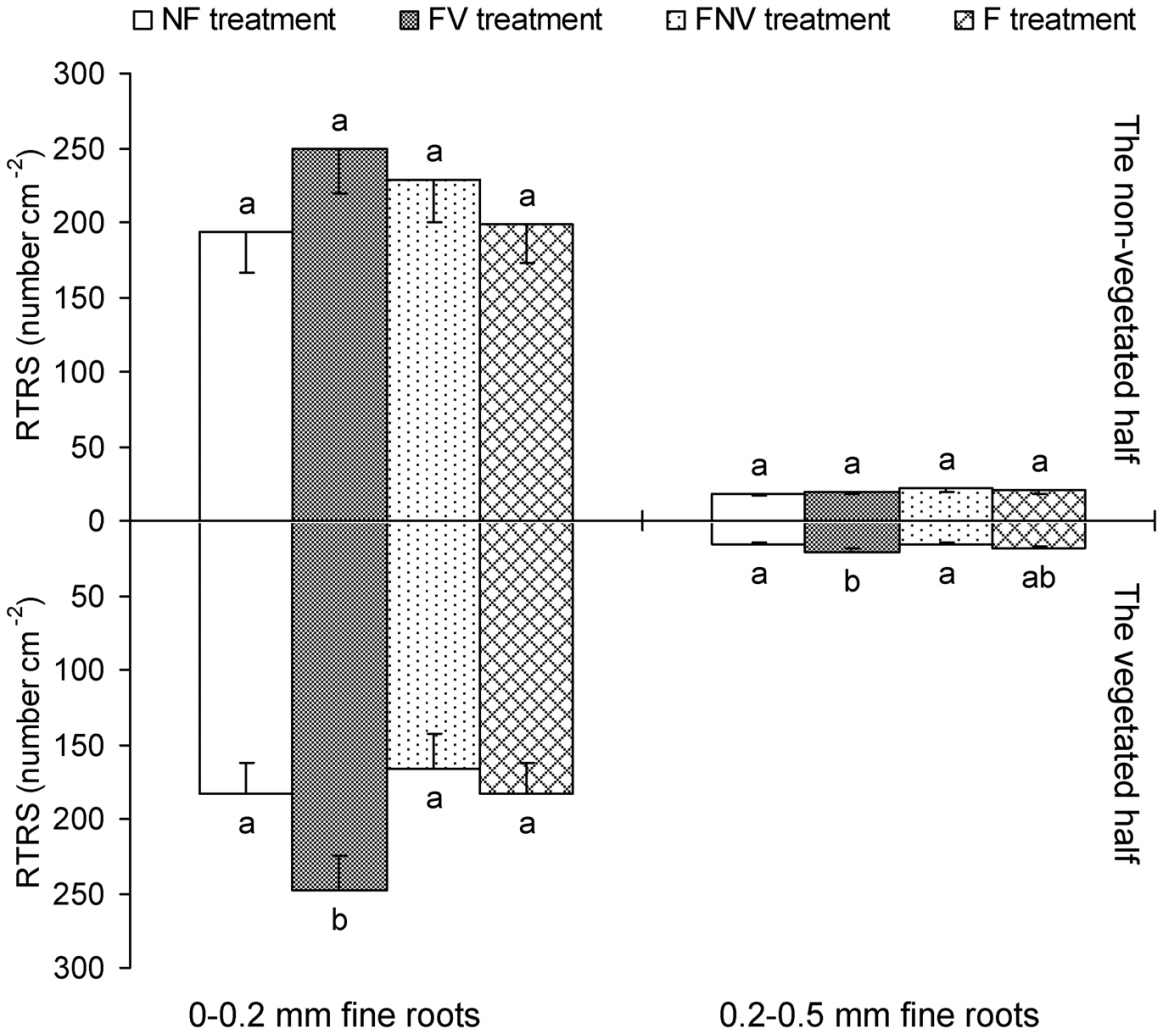
The number of root tips over root surface (RTRS), root architecture indicator, in the vegetated half and in the non-vegetated half. Letters indicate the same subclass (0–0.2 mm or 0.2–0.5 mm fine roots) difference between treatments (LSD tests, following ANOVA). Error bars represent 1 SE of the mean.

RTRS of both 0–0.2 mm and 0.2–0.5 mm fine roots in the non-vegetated half was shown to be significantly higher than that in the vegetated half for FNV treatment (i.e. ratio less than 1), and no difference was found in the other treatments (Fig. 2). In the fertilization of the non-vegetated half for FNV treatment, the target plants increased spatial nutrient uptake by altering RTRS. In the vegetated half, RTRS of 0–0.2 mm fine roots for the FV treatment was significantly higher compared with the other three treatments, reaching a maximum of 247.7 cm^−2^. RTRS of 0.2–0.5 mm fine roots for the FV treatment was also higher than those obtained in the NF and FNV treatments, with values of 20.3, 15.6, and 16.1 cm^−2^, respectively (Fig. 4). Since the RTRS of 0–0.2 mm fine roots was much higher than that of 0.2–0.5 mm fine roots in all of the four treatments, RTRS of the latter had little effects on root foraging ability compared with the former. The RTRS of 0–0.2 mm fine roots in the vegetated half significantly increased from 182.8 cm^−2^ in the NF treatment to 247.7 cm^−2^ in the FV treatment, and significantly decreased to 182.6 cm^−2^ in the F treatment. In addition, there was no significant difference in the RTRS values of both fine root subclasses among all the four treatments in the vegetated half (Fig. 4).

### Root Architecture Indicator: SRLP

**Figure 5:**
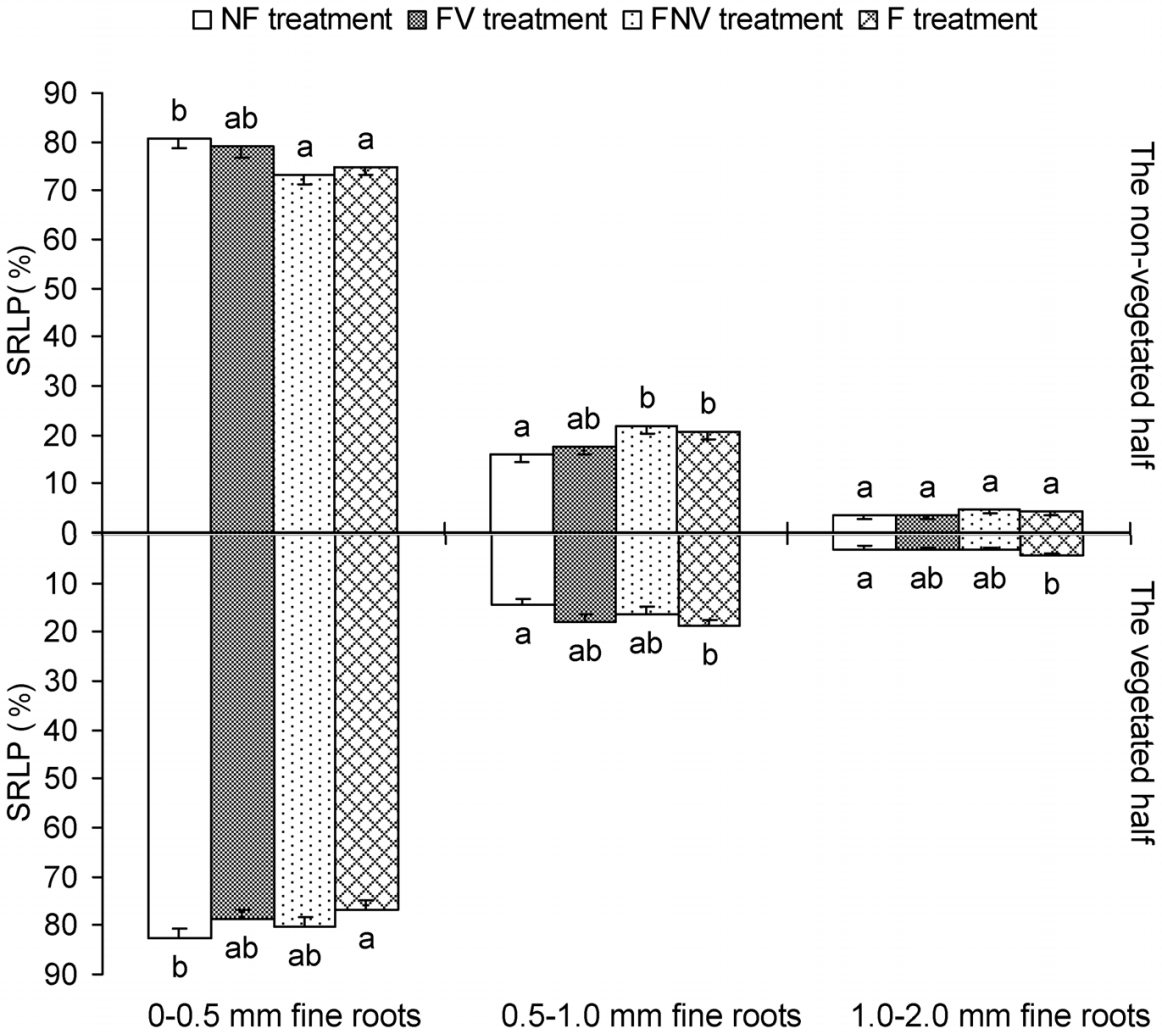
The length percentage of diameter-based fine root subclasses to the total fine root length (subclass root length percentage, SRLP), root architecture indicator, in the vegetated half and in the non-vegetated half. Letters indicate the same subclass (0–0.5 mm, 0.5–1.0 mm or 1.0–2.0 mm fine roots) difference between treatments (LSD tests, following ANOVA). Error bars represent 1 SE of the mean.

The 0–0.5 mm root systems mainly consisted of the first three orders; the SRLP of which may reflect length proportion of the root systems being able to absorb nutrient and water in the soil to whole fine root. 0–0.5 mm fine root in the FNV treatment had significantly higher SRLP ratios (i.e. the ratio was significantly more than 1), 0.5–1.0 mm fine roots had lower SRLP ratios (i.e. less than 1), whereas no differences were found between the vegetated and non-vegetated halves in all the other three treatments (Fig. 2). The significantly higher SRLP ratio of 0–0.5 mm fine roots in the FNV treatment indicated the target plant’s attempt to strengthen nutrient acquisition in the observed space. The SRLP of 0–0.5 mm fine roots in the F treatment was significantly lower in the vegetated and non-vegetated halves, as compared with that of the NF treatment. The opposite trend was found for the SRLP of 0.5–1.0 mm fine roots (Fig. 5). The lower SRLP of 0–0.5 mm fine roots helped reduce the absorbing root length density, thereby alleviating root competition intensity within the same plant root system.

### Root Architecture Indicator: ROLP

**Figure 6:**
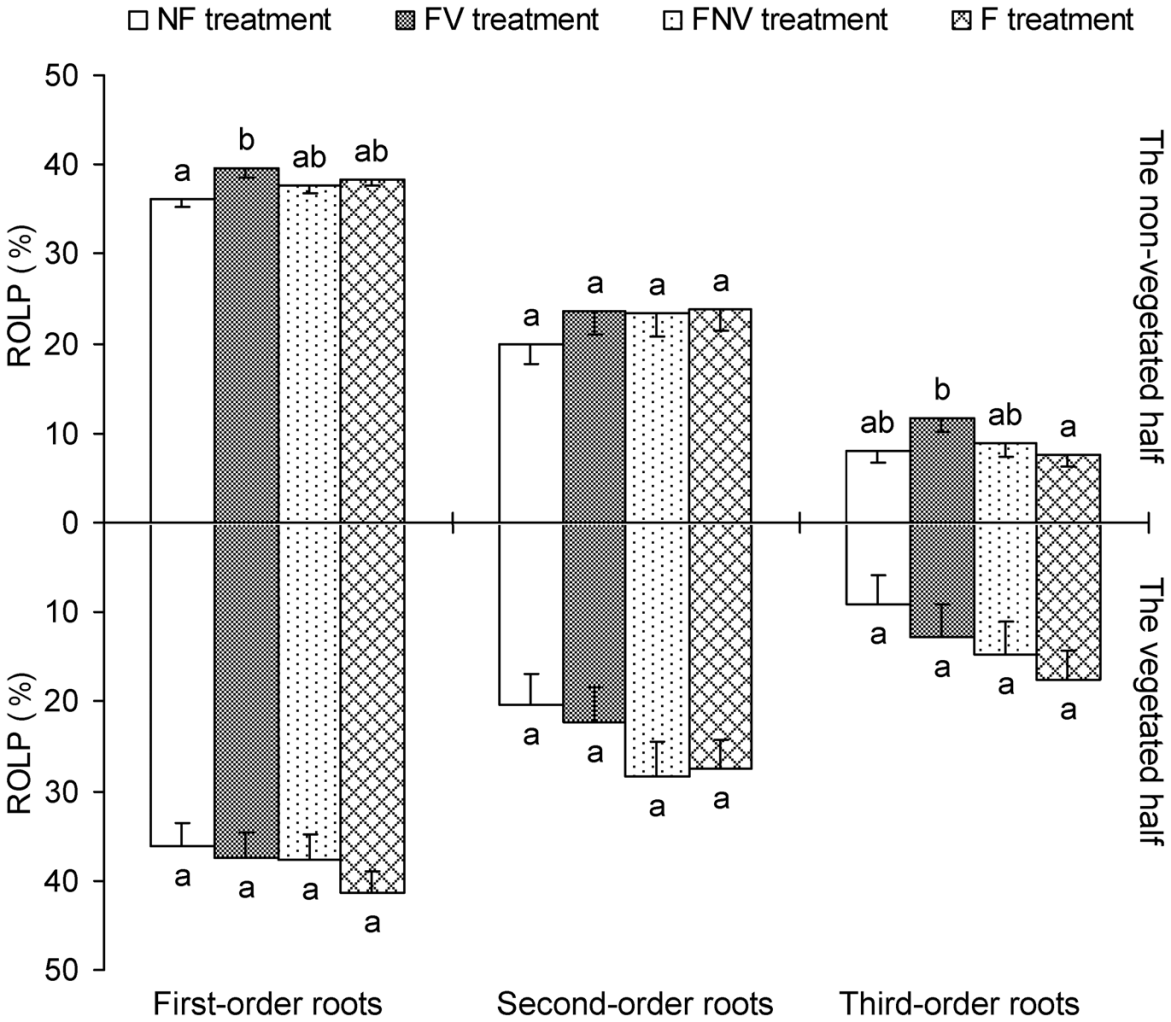
The length percentage of each root order to the total fine root length (root order length percentage, ROLP), root architecture indicator, in the vegetated half and in the non-vegetated half. Letters indicate the same root order difference between treatments (LSD tests, following ANOVA). Error bars represent 1 SE of the mean.

The length percentages of the first three root orders against the total fine root contributed to the further analysis of the inner changes in the SRLP of 0–0.5 mm fine roots, as mentioned above. The third-order ROLP ratio in the FNV treatment was significantly more than one (Fig. 2), which explained the higher SRLP ratio of the 0–0.5 mm fine roots in the FNV treatment. The ROLP of the first-order roots in the non-vegetated half with the FV treatment was significantly higher than in those with the NF treatment. The ROLP in the non-vegetated half of the third-order roots in the FV treatment were likewise higher than in the F treatment (Fig. 3). However, no significant differences in the ROLP values of the three root orders were found among all the four treatments (Fig. 6).

### The Relative Growth Rate (RGR)

**Figure 7:**
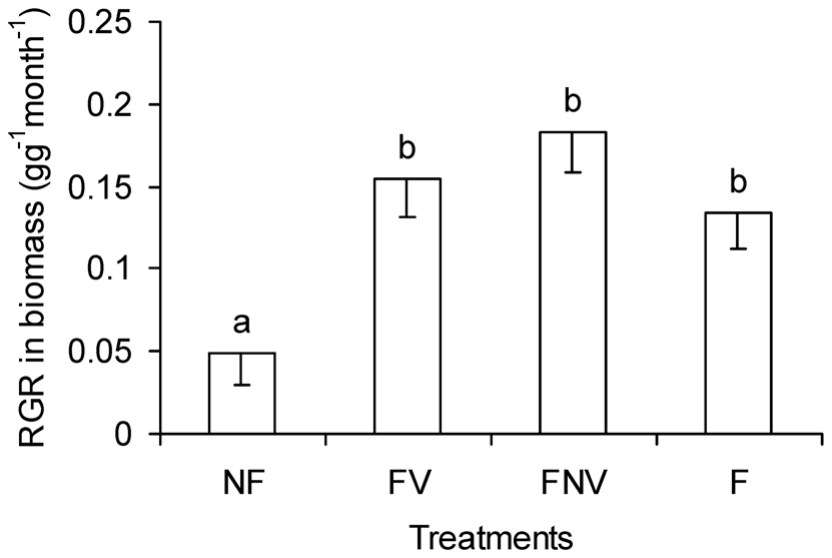
The relative growth rate (RGR) of target plant in different treatments. Letters indicate RGR differences between treatments (LSD tests, following ANOVA). Error bars represent 1 SE of the mean.

The RGR of the FV and FNV treatments were significantly higher, as compared with that in NF treatment, but were not significantly different from the F treatment (Fig. 7). Given that the total amount of nutrients used in the FV and FNV treatment was half of that in the F treatment, the absence of significant differences between both treatments indicated that the target plants, which were simultaneously exposed to nutrient heterogeneity and the roots of the neighboring plants, had excellent nutrient uptake abilities.

## Discussion

De Kroon et al. proposed that the interplay between local responses and systemic modifications of these responses was an essential feature of plant foraging. More specifically, plant foraging for resources was achieved through various processes acting in concert at the level of the repetitive units (modules), from which plant roots were constructed. These processes involved individual module responses to localized environmental signals and the systemic control of these responses. This systemic control can be achieved by the signals received from connected modules exposed to different conditions or by those reflecting the overall resource status of the plant [2]. However, past evidence of the interplay between local responses and systemic modifications in plant root foraging behavior was limited. In the present study, the significant increase in the RTRS of 0–0.2 mm fine roots in the vegetated half occurred from the NF treatment (without fertilizers) to the FV treatment (with fertilizers only in the vegetated half); the significant decrease in the RTRS of the 0–0.2 mm fine roots in the vegetated half was observed from the FV to the F treatments (i.e., nutrients are supplemented in both the vegetated and non-vegetated halves, as compared to FV treatment), with the conversion of the whole plant root resource status from the localized nutrient supply in the FV treatment (i.e., fertilizer only in vegetated half) to the overall nutrient supply in the F treatment (i.e., fertilizer in both halves). These results clearly showed that RTRS was regulated by the local responses and the systemic controlled mechanisms. For the first time, our findings provided new evidence based on root architecture for De Kroon’s concept, which directly reflect the root foraging ability of woody plants.

Cahill et al. hypothesized that plants integrate information from both resource and neighbor-based cues in the environment in a non-additive manner [8]. However, they measured the horizontal spread of the roots, which was unsuitable for precisely exploring the root foraging ability, as compared with the root biomass or architecture. In our study, the 0–0.5 mm fine roots SRLP of both the vegetated and non-vegetated halves decreased with the increasing nutrient concentrations, based on the results of the NF and F treatments. Therefore, the target plant adopted strategies to ease the competition within the same plant root system as the nutrient status increased. The RTRS of the vegetated half and the ROLP of the first-order roots in the non-vegetated half were higher in the FV treatment than in other treatments. In addition, these indicators were significantly different between the vegetated and non-vegetated halves in the FNV treatment. Collectively, we were able to show that plants used novel root foraging behaviors under different combinations of environmental conditions, such as neighboring plants and localized fertilization. We took full advantage of the root architecture indicators to effectively measure foraging behaviors and provide pronounced evidence that woody plant root foraging behavior was a non-additive response to multiple forms of environmental information.

When grown in heterogeneous conditions, plants preferentially produce roots in nutrient-rich substrate patches, and enhance the uptake efficiency of these roots, as compared with other roots of the same plant outside the patch zone [38], [46]. The differences between the NF and FV treatments indicated that the target plants increased their nutrient uptake in nutrient-rich patches by altering the root architecture (RTRS) under the conditions of constant absorbing root biomass. Despite the intense competition in the same patches, root competition did not affect the attempts of plants to absorb resources in nutrient-rich patches. In addition, the RTRS ratio in the FNV treatment was less than 1, which reflected the attempt of the target plants to strengthen the nutrient intake in nutrient-rich patches. Mommer et al. suggested that the root response to nutrient distribution in a competitive environment depended on the competitive strength of the neighboring species; in their study, competition with a superior competitor led the inferior *Agrostis stolonifera* to increase relative root investment in the nutrient-poor patch instead of the nutrient-rich patch [10]. Under similar competitive strength conditions by neighboring species (i.e., intraspecific competition), the target plants in the present study still had enhanced nutrient uptake in the nutrient-rich patches, which showed that plants seemed to prefer nutrient intake in nutrient-rich patches than in the nutrient-poor counterparts unless forced by enormous environmental stress, such as competition with more superior competitor (with larger competitive advantage). Therefore, the unit cost of soil resource acquisition was lower in the nutrient-rich patches than in the nutrient-poor ones.

Some plants may engage in a game of “Tragedy of the Commons” when competing for soil resources. Thus, a plant in the presence of neighboring roots should preferentially place new roots in unoccupied soil instead of the space containing roots of other species or conspecifics [21], [39]. The target plant in the FV treatment had a higher ROLP and biomass for the first root order in the non-vegetated half, as compared with the NF treatment; higher ROLP was observed in the third-order roots of the non-vegetated half with the FV treatment, as compared with the F treatment. Despite the lower soil resource concentration in the non-vegetated half than in the vegetated one, the plant still attempted to increase the nutrient intake in this space. Furthermore, the plants intensified nutrient uptake in the non-vegetated half by altering the RTRS in FNV treatment, as described above. Therefore, plants simultaneously exposed to nutrient heterogeneity and neighboring plants still attempted to increase nutrient uptake in the space free of other plant roots, regardless of the distribution of resources.

The non-additive root growth response under the combined nutrients and neighbors environments (i.e. interactions occur) may be due to the interplay between local responses and systemic modifications of the response. When intense competitive signals were received from the connected modules (i.e., roots in the vegetated half) in the FV treatment, the target plants increased their nutrient uptake in the non-vegetated half by investing more first-order root biomass and increasing the ROLP of first-order roots in the non-vegetated half, as compared with the NF treatment, and by increasing the ROLP of the third-order roots in the FV treatment, as compared with the F treatment. Because the fraction of nutrients obtained from the non-vegetated half to nutrients the whole plant desired was increased, the intense competition in the vegetated half was alleviated in the FV treatment. In other words, target plants increasing their nutrient uptake in the non-vegetated half helped decrease the fraction of nutrients obtained from the vegetated half. The interaction between roots in the different halves (modules) triggered potential nutrient uptake ability of whole plant root system, with more powerful nutrient uptake observed in both non-vegetated and vegetated halves. Although facilitators of soil resource acquisition were present in the non-vegetated half, as well as higher nutrient concentrations and the absence of interspecific root competition, the target plants in the FNV treatment still increased their nutrient uptake in the vegetated half than in the non-vegetated one, with higher SRLP in the 0–0.5 mm fine roots and higher ROLP of the third-order roots. Therefore, competition was strengthened in the vegetated half, based on the interplay between the local responses and systemic controls. This response was necessary for late-succession trees to be established in fully occupied belowground environments to ensure long-term success of the said tree population. Given the similar nutrient concentration between two halves in the NF and F treatments, induction of root growth in nutrient-rich patches was lost and root competition became the most important environmental stimulus. That is, our study indicated that under the combinations of homogeneous nutrients and root competition, target plants adopted the strategies of deceasing SRLP in 0–0.5 mm fine roots, either in the non-vegetated or vegetated halves, to alleviate inter- and intra-plant root competition with the increasing nutrient concentration. The lower SRLP in 0–0.5 mm fine roots (the significant region in nutrient absorption) contributed to mitigate intra-plant root competition because competition among roots of the same plant was three- to five-times greater than competition among roots of neighbouring plants [47]. Collectively, the interplay between the local responses and the systemic response modifications in root foraging was far more complicated under a combination of neighboring competitors and nutrient heterogeneity than that of neighboring competitors and homogeneous nutrient conditions. The sophisticated interaction between local response and systemic control originated from the existing nutrient differences and neighboring plant roots, which triggered the potential root foraging ability under a combination of neighboring competitors and nutrient heterogeneity. This phenomenon may account for the similar relative growth rate (RGR) among the plants in the FV, FNV, and F treatments.

In this study, contrary to the small biomass difference in the first three root orders between different treatments, root architecture indicators that originated from these root systems were greatly varied. Therefore, the root architecture responded to environmental stimuli more sensitively than the root biomass. Moreover, the plant’s attempt to increase nutrient uptake was reflected by the altered root architecture but with constant biomass. Given that the roots possessing essential nutrient uptake ability represent only a portion of the entire root system for woody plants, the root architecture indicators constructed by these roots (i.e., the first three root orders or the 0–0.5 mm roots in diameter) in our study were more precisely measured the root foraging ability, as compared with the methods used in previous investigations. These root architecture indicators provided us with a novel and effective means to explore woody plant root foraging behavior.
